# Segmentor: a tool for manual refinement of 3D microscopy annotations

**DOI:** 10.1186/s12859-021-04202-8

**Published:** 2021-05-22

**Authors:** David Borland, Carolyn M. McCormick, Niyanta K. Patel, Oleh Krupa, Jessica T. Mory, Alvaro A. Beltran, Tala M. Farah, Carla F. Escobar-Tomlienovich, Sydney S. Olson, Minjeong Kim, Guorong Wu, Jason L. Stein

**Affiliations:** 1 RENCI, University of North Carolina at Chapel Hill, 100 Europa Drive, Suite 540, Chapel Hill, NC 27517 USA; 2 UNC Neuroscience Center, University of North Carolina at Chapel Hill, 116 Manning Drive, CB# 7250, Chapel Hill, NC 27599 USA; 3 Department of Genetics, University of North Carolina at Chapel Hill, Chapel Hill, NC 27599 USA; 4 Department of Computer Science, University of North Carolina at Greensboro, Greensboro, NC 27412 USA; 5 Department of Psychiatry, University of North Carolina at Chapel Hill, 334 Emergency Room Drive, 343 Medical Wing C, Chapel Hill, NC 27599 USA; 6 Department of Computer Science, University of North Carolina at Chapel Hill, Chapel Hill, NC 27599 USA

**Keywords:** Tissue clearing, Light sheet microscopy, Deep learning, Image segmentation, Manual annotation

## Abstract

**Background:**

Recent advances in tissue clearing techniques, combined with high-speed image acquisition through light sheet microscopy, enable rapid three-dimensional (3D) imaging of biological specimens, such as whole mouse brains, in a matter of hours. Quantitative analysis of such 3D images can help us understand how changes in brain structure lead to differences in behavior or cognition, but distinguishing densely packed features of interest, such as nuclei, from background can be challenging. Recent deep learning-based nuclear segmentation algorithms show great promise for automated segmentation, but require large numbers of accurate manually labeled nuclei as training data.

**Results:**

We present Segmentor, an open-source tool for reliable, efficient, and user-friendly manual annotation and refinement of objects (e.g., nuclei) within 3D light sheet microscopy images. Segmentor employs a hybrid 2D-3D approach for visualizing and segmenting objects and contains features for automatic region splitting, designed specifically for streamlining the process of 3D segmentation of nuclei. We show that editing simultaneously in 2D and 3D using Segmentor significantly decreases time spent on manual annotations without affecting accuracy as compared to editing the same set of images with only 2D capabilities.

**Conclusions:**

Segmentor is a tool for increased efficiency of manual annotation and refinement of 3D objects that can be used to train deep learning segmentation algorithms, and is available at https://www.nucleininja.org/ and https://github.com/RENCI/Segmentor.

**Supplementary Information:**

The online version contains supplementary material available at 10.1186/s12859-021-04202-8.

## Background

The structure of the brain provides the machinery that enables behavior and cognition. The human brain is extremely complex, comprising ~170 billion cells, of which ~86 billion are neurons. The mouse brain, a common model system used to study brain-behavior relationships, is much smaller yet still has ~109 million cells, ~70 million of which are neurons [1]. By mapping the location of these many brain cells, classifying them into types based on the expression of marker genes, and determining how cell type proportions and locations are altered by mutations or environmental factors, we can understand how changes in brain structure lead to changes in behavior and/or cognition.

In order to map cell types within intact brains or any other tissue, a number of tissue clearing techniques for making tissues transparent were recently introduced [2, 3]. Combined with high-speed image acquisition through light sheet microscopy, the full 3D extent of adult mouse brain specimens can be imaged at micron resolution in a matter of hours [4–7].

Currently, these large-scale microscopy images are often used for qualitative visualization rather than quantitative evaluation of brain structure, thus potentially overlooking key spatial information that may influence structure–function relationships for behavior and cognition. In order to quantify objects within annotated regions of the images from the brain or any other tissue, we need to distinguish morphological objects of interest (e.g., nuclei) from background [8]. Existing programs that perform object segmentation in cleared samples from tissue (for example, ClearMap [9], CUBIC [10]) or organoids [11] work well for cases with unambiguous morphological characteristics. However, for cases in which morphological objects are densely packed, nuclei segmentation results are less accurate using current computational tools, which obfuscates brain structure quantifications and comparisons. Recent deep learning-based nuclear segmentation algorithms such as multi-level convolutional neural networks show great promise for more accurately identifying each individual nucleus [12–15]. When colocalized with immunolabeling, nuclear segmentation additionally enables counting individual cell types. Present learning-based methods require two sets of manually labeled ‘gold standards’: (1) a large number of training objects to learn the morphometrical appearance of nuclei in the context of various backgrounds, and (2) independent benchmark datasets for evaluating the accuracy of automated segmentation results.

Gold standard datasets are derived from manual labels by trained and reliable raters. Manual labeling is both time-consuming and difficult because of ambiguities in nuclear boundaries and the inherent challenges of labeling 3D structures on a 2D screen. A few tools have been developed for manual labeling of objects in 2D [16] and 3D [17–23] images, including labeling in virtual reality environments [24]. Existing tools are generally optimized for segmenting objects derived from specific image modalities. For example, VAST [21] and WebKnossos [23] are optimized for electron microscopy imaging where subcellular compartments can be resolved, and therefore enables hierarchical parent–child object relationships to be specified for segmentations, such as dendritic spines from dendritic arbors from a given neuron. Here, we focus on developing a new tool optimized for manual annotation of densely packed nuclei within large images derived from tissue clearing and fluorescence microscopy. In these images, the high visual complexity due to overlapping and/or neighboring nuclei boundaries within a 3D scene makes accurate segmentation challenging. We specifically focused on implementing features that enhance the efficiency of this task, including synchronized 2D + 3D visualization and editing, intuitive visibility controls, and semi-automated correction of segmentation errors common to light sheet microscopy of brain tissue (e.g., incorrectly merged or split nuclear boundaries).

We present Segmentor, an open-source tool for reliable, efficient, and user-friendly manual annotation and refinement of objects (e.g., nuclei) within 3D light sheet microscopy images from any tissue. This tool enables automated pre-segmentation of nuclei, refinement of objects in 2D and 3D, visualization of each individual nucleus in a dense field, and semi-automated splitting and merging operations, among many other features. Table 1 provides a feature-based comparison between Segmentor and four other tools that enable manual annotation of objects within 3D imaging data (VAST Lite [21], Labkit [22], Brainsuite [20], and webKnossos [23]). Segmentor has been used by 20 individuals to achieve reliable segmentation and labeling of thousands of nuclei. We show results following a case study that editing simultaneously in both 2D and 3D significantly decreases labeling time, without impacting accuracy, as compared to the user being presented the same set of images with 2D editing capabilities only. Software releases of this tool and an example image are available at https://www.nucleininja.org/, and source code and documentation are available at https://github.com/RENCI/Segmentor. We expect that increasing the number of manually labeled nuclei in 3D microscopy images through this user-efficient tool will help implement fully automated data-driven nuclear recognition via deep learning approaches.

**Table 1:** Comparison of features among Segmentor and three other tools [20–23] that enable manual editing of segmentation volumes

	Segmentor 0.3.2	VAST Lite 1.4.0	Labkit 0.2.6	Brainsuite v19.b	webKnossos
Available packages	Windows, Mac, Linux	Windows	Windows, Mac, Linux	Windows, Mac, Linux	Web-based
Image File Format	.nii,.vti,.tiff (single and multistack)	.vsv,.vsvol,.vsvi,.vsvr	.tiff	.img,.img.gz,.nii,.nii.gz	.czi,.nii,.raw,.dm3,.dm4,.png,.tiff (single and multistack)
Segmentation file format	.nii,.vti,.tiff (single and multistack)	.vss,.vsseg	.tiff,.h5	.nii.gz	.stl
2D + 3D editing	Yes	No	No	No	No
Synchronized 2D + 3D views	Yes	No	No	No	Yes
3D visibility controls for densely packed objects	Yes	Yes	No	No	Yes
Voxel-level editing	Yes	Yes	Yes	Yes	Yes
Region-level controls (e.g., merge/split)	Yes	Yes	No	No	Yes
Hierarchical object relationships	No	Yes	No	No	Yes
Source code available	Yes	No	Yes	Yes	Yes

### Implementation

#### Software

**Fig. 1:**
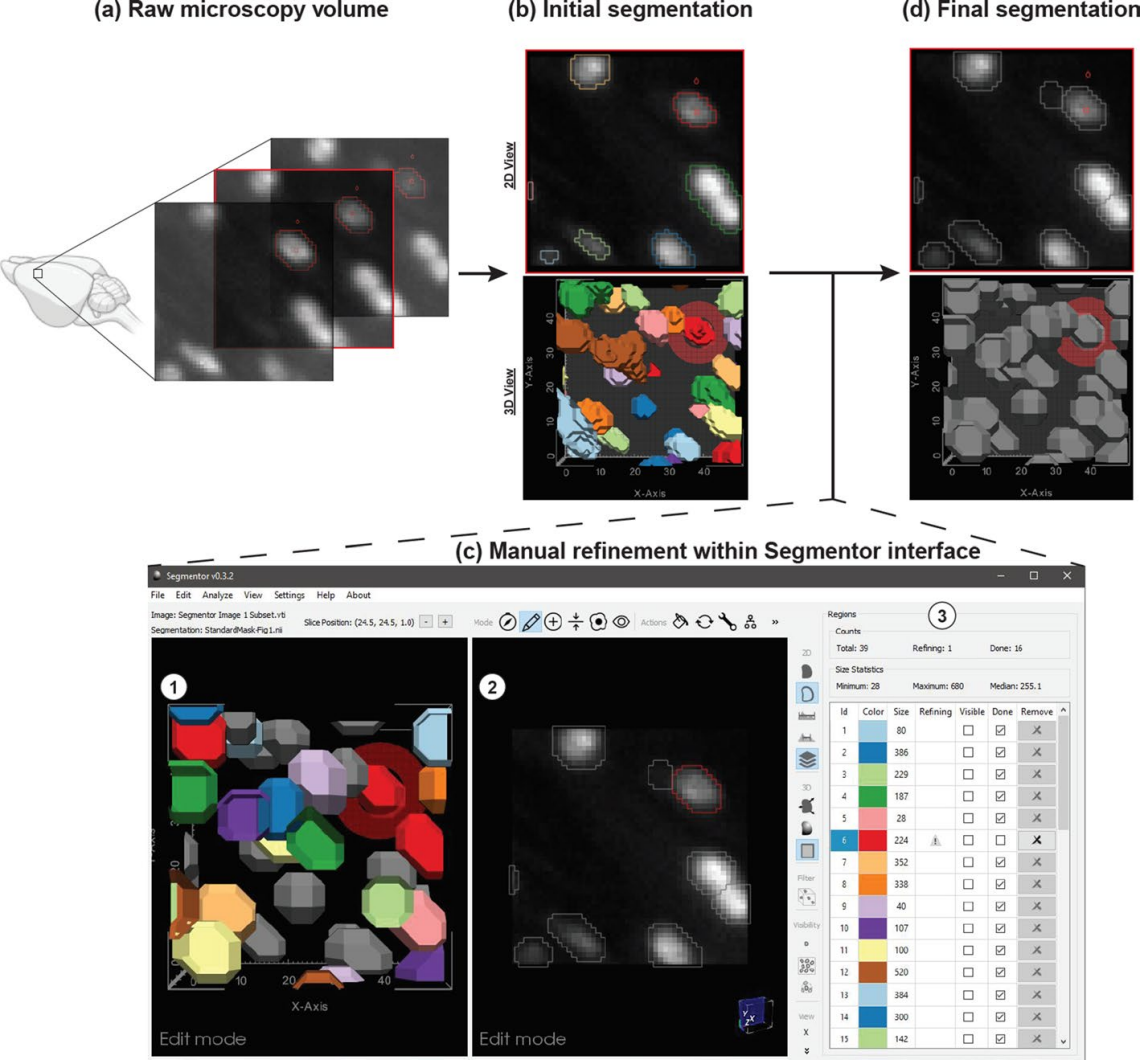
Demonstration of Segmentor software for nuclear refinement. **a** Raw microscopy volumes of the brain are loaded into the software. **b** Segmentor provides an initial segmentation of nuclei within the image (alternatively, pre-segmentations from other programs can be loaded). **c** The segmented images are manually refined within Segmentor using (1) the 3D visualization of segmented nuclei and (2) the 2D slices. (3) The region table enables the user to track progress during segmentation. **d** Finally, the manually refined image that can be used as gold standard input to deep learning programs is shown (grey regions indicate those the user has marked as completed). Image made in part using BioRender

The Segmentor tool was developed in C++ using open-source cross-platform libraries, including VTK [25] and Qt [26]. 3D image volumes and segmentation data can be loaded in TIFF, NIfTI, or VTI format. To increase efficiency, the tool is primarily designed for manually refining existing annotations rather than beginning annotations completely anew. The user can load initial segmentation data generated by a tool external to Segmentor (e.g., NuMorph [12], CUBIC [10], or ClearMap [9]), or generate an initial global intensity threshold-based segmentation [27] from within Segmentor. The interface consists of panels with 2D (right panel) and 3D (left panel) views and a region table (Fig. 1). The 2D view consists of a single slice through the volume and enables the user to see both the voxel intensities as well as 2D visualizations of the segmented regions. The 3D view enables the user to see 3D surfaces of the segmented regions and inspect them for non-uniform morphology that is difficult to visualize using only the 2D view. The 2D and 3D views are synchronized such that navigating (i.e., rotating, translating, or zooming) in one view updates the other view simultaneously. The hybrid 2D + 3D visualization and editing capabilities are important as each view offers multidimensional context for the annotation procedure, e.g., the 2D view is useful for manually selecting voxels based on image intensity, whereas the 3D view is useful for identifying incorrectly segmented regions of densely packed nuclei. This feature, to our knowledge, is not found in other existing image annotation software to date (Table 1).

#### Visualization features

The 2D view provides outline and solid overlay representations of the segmented regions, and window/level controls for the voxel intensities. The 3D view has controls for smooth shading and surface smoothing. The user can also toggle a representation of the current 2D slice plane in the 3D view. Edits made in either view are immediately updated in the other view. Various modes were designed to enhance the ability to annotate in the 3D view and render 2D + 3D scene information, including 1) the current slice plane, 2) the currently selected region and close neighbors, and 3) the currently selected region only.

#### Editing features

Various editing features are provided. Most operations can be applied in either the 2D or 3D view, although certain features are only applied in the 2D X–Y plane due to the improved resolution in that plane for most microscopy volumes. Standard editing features include voxel-level painting and erasing of the currently selected region. The user can select a customizable brush radius, applied in the X–Y plane, for these edits.

In addition to these standard editing features, more advanced features are also provided. The user can apply a constrained region growing or shrinking operation in the X–Y plane by selecting a voxel outside (growing) or inside (shrinking) the current region. For region growing, all voxels with an intensity equal to or higher than the selected voxel that are reachable from the current region, and no farther than the selected voxel, are added to the region. This is similar to a dilation of the current region, but only including voxels with intensities greater than or equal to the current region. Region shrinking works similarly, but removes voxels with intensities less than or equal to the selected voxel.

**Fig. 2:**
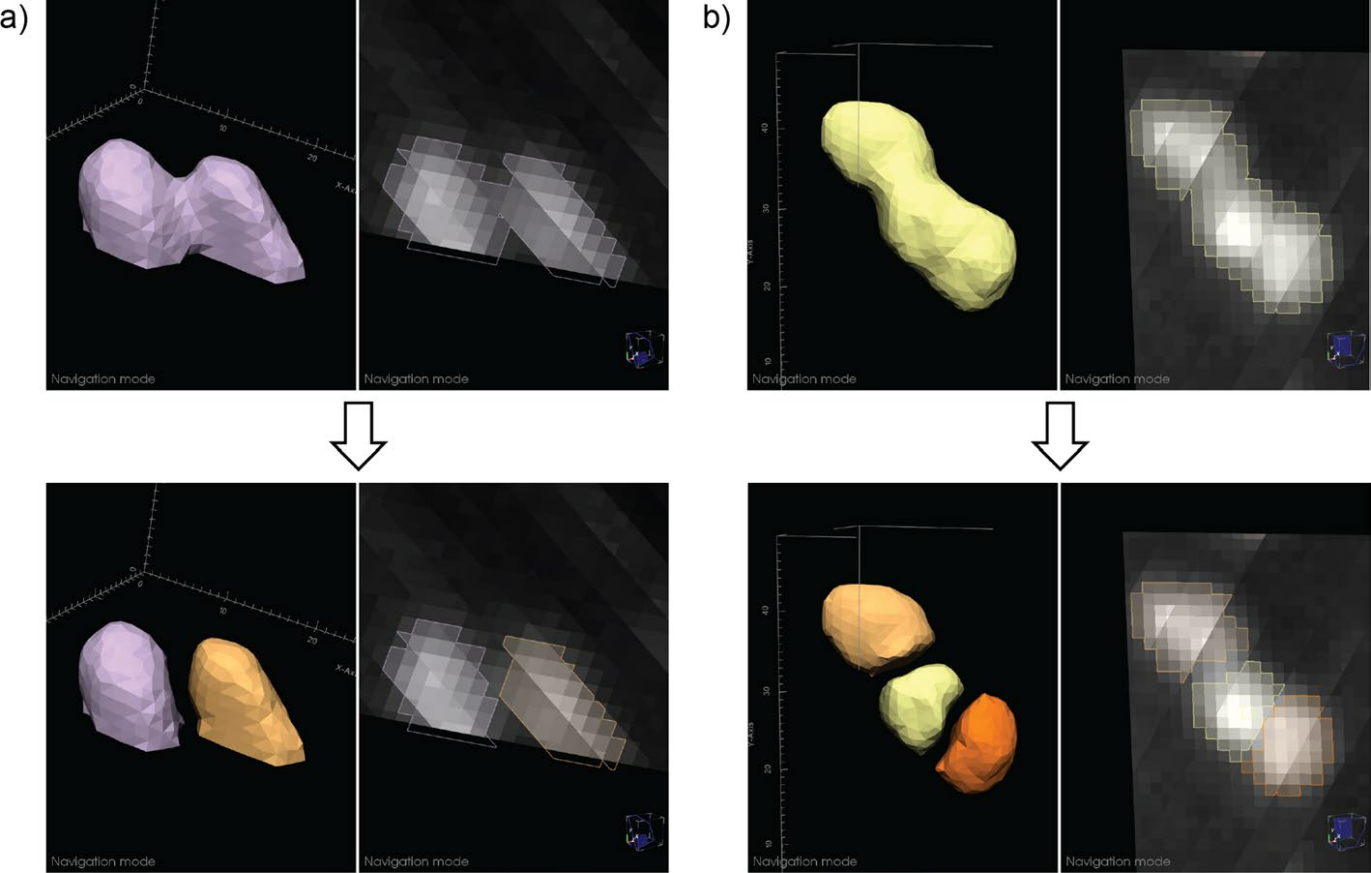
Examples of automated nuclear splitting within Segmentor. **a** An incorrectly joined region is shown (top), which after visual inspection is determined to represent two nuclei. After the user specifies that there are two nuclei in the joined region, the automated splitting function result is shown (bottom). **b** Similar to (**a**), but three nuclei are incorrectly joined (top) and the automated result is shown (bottom)

Common segmentation problems from automatic methods include divided nuclei, when more than one region is present within a single nucleus, and joined regions, when multiple nuclei are incorrectly included as the same region. Semi-automated methods are provided for correcting these issues. To fix divided nuclei, the user can select any region to merge with the current region by reassigning the voxel labels. Splitting joined regions is more challenging (Fig. 2). We employ an intensity threshold method: using the 2D and 3D views, the user determines how many nuclei are in the current region that should be separated. After specifying this number, a fully-automated approach is applied. An increasing intensity threshold is repeatedly applied to the voxels in the region. As the intensity increases, the region is typically broken up into smaller regions. The threshold resulting in the specified number of regions (via connected component analysis) with the largest volume for the smallest of the three regions (making the method less sensitive to noise) is used to define seed regions (intensities are typically higher toward the center of the nuclei). Each seed region is then successively grown similarly to the region growing method described above, by stepping the region growing intensity down from the seed region threshold, constraining the growing to a 1-voxel radius at each step, and to the original region voxels. After splitting, the user can perform any necessary adjustments using the other editing features.

#### Region table

To help the user manage the complexity of segmenting many nuclei in a given volume (e.g. ~ 460 nuclei are found within a typical image volume of 96 μm × 96 μm × 160 μm of the adult mouse cortex that we use for manual labeling), a region table provides information on each segmentation region, including label color, size (in voxels), modified status (whether the label has been modified since the last save), and done status (whether the user considers segmentation complete for that region). The user can sort by label, size, or status, and select any region to zoom in on that region in the 2D and 3D views. The user can mark any region as done to keep track of their progress. Such regions will be greyed out in the other views. Modified and done statuses are stored in a separate JSON metadata file stored with the segmentation data.

#### Typical workflow

All users undergo an initial training period in which they receive the same standardized training image containing 39 nuclei. Each user then generates an initial automated segmentation, which s/he manually edits. Labeling reliability is then iteratively assessed by comparing segmentations to those of an experienced rater (CMM) until a Dice score [28] of ≥ 0.85 is achieved and label counts are within ± 1 nucleus of the ‘gold standard’ training segmentation (i.e., 39 ± 1 nuclei).

#### Case study

To quantify the efficiency and accuracy of manual labeling in 2D + 3D as compared to 2D alone, two users (CMM, NKP) manually refined a series of four images using either ‘2D only’ or synchronized ‘2D + 3D’ visualization and editing. Both annotators used Segmentor v0.2.11 (Windows version) and achieved reliability on a separate standardized image prior to beginning the case study. One user (NKP) was assigned these 4 images balanced with respect to the order of ‘2D only’ or ‘2D + 3D’, to minimize ordering bias. This user alternated between ‘2D only’ and ‘2D + 3D’ using a toggle-enabled feature in Segmentor’s interface designed to hide the 3D visualization. In total, this user completed 2 manual refinements on each of the 4 images (i.e., labeling the same image twice per visualization modality). The other user (CMM) edited each of the four images in ‘2D + 3D’ only for accuracy assessment. Both users recorded the time to completion using the freely available Clockify application. Manually refined annotations were compared between users for accuracy (Dice score), and differences in time and accuracy between ‘2D only’ and ‘2D + 3D’ were evaluated using two-tailed paired t-tests in R.

#### Image acquisition

Images were acquired from iDISCO + tissue clearing [9] of postnatal day 15 (P15) C57Bl/6 J mice. Nuclei were labeled with TO-PRO-3 and imaged on a light sheet microscope (Ultramicroscope II from LaVision Biotec) at a final resolution of 0.75 μm × 0.75 μm × 2.50 μm. Blocks from the cortex were used for labeling with Segmentor. Further details about image acquisition can be found in [12].

#### User survey

Six users consented to provide Segmentor usability feedback in a 28-question survey (24 Likert scale questions on a 7-point scale, in which ‘1’ means ‘not useful’ and ‘7’ means ‘extremely useful,’ followed by 4 open-ended questions). Qualtrics^XM^ was used to distribute the survey and analyze participant results (see Additional File 1).

## Results

Twenty users have used Segmentor for manual refinement of 3D microscopy volumes. Segmentations from one expert user were defined as the gold standard and results from every other user were compared to this segmentation via Dice score and nuclei counting to assess reliability. After several iterations, a Dice score of ≥ 0.85 was achieved by each user (average final Dice score = 0.885). We did not quantify the intra-operator agreement, but we expect that it would be higher than the inter-operator agreement.

**Fig. 3:**
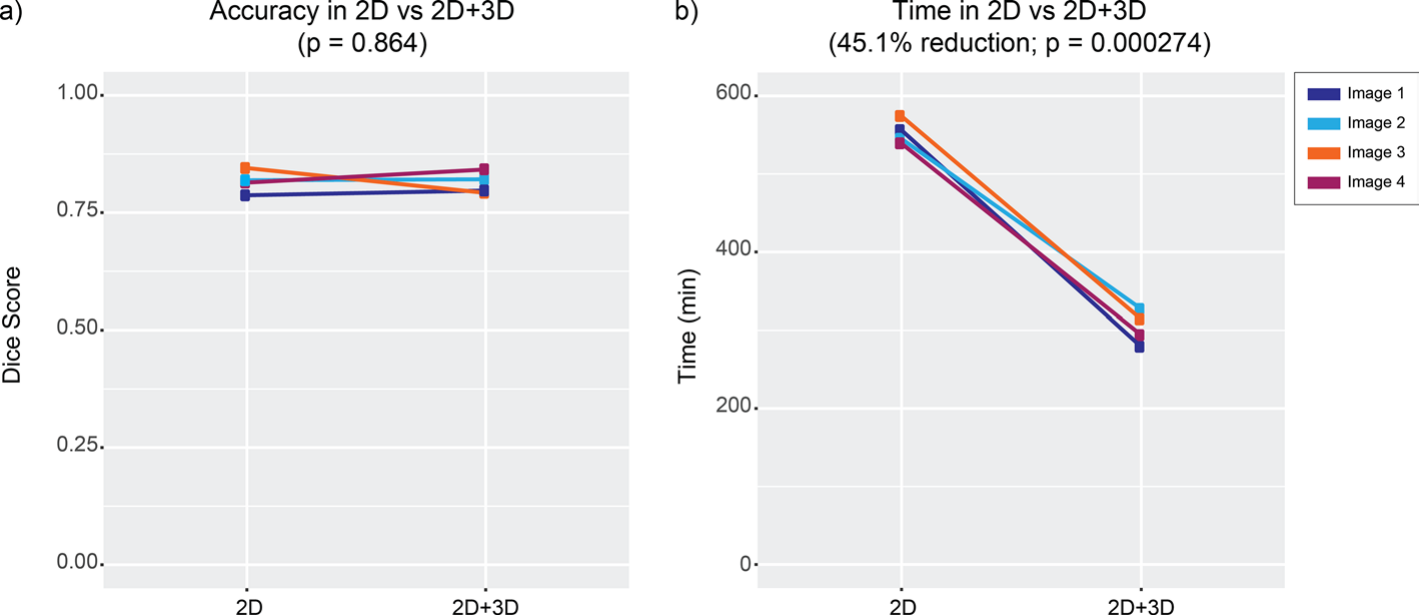
Results of case study to determine accuracy and efficiency of manual refinement when editing and visualizing in 2D only vs. 2D + 3D. **a** Dice score measuring accuracy relative to an expert rater for either the labels only from the 2D segmentations or from 2D + 3D segmentations. **b** Time comparison between 2D vs 2D + 3D showing a 45.1% reduction to manually refine nuclei (*p* = 0.00027)

We designed a case study to test the impact of simultaneous visualization and editing in 2D and 3D on manual labeling efficiency and accuracy. One user labeled nuclei in 4 images containing ~ 39 nuclei, using either the 2D view alone with the 3D view and editing features turned off, or using the synchronous 2D and 3D views and editing features. A separate expert user annotated the same images with both the 2D and 3D views to serve as gold standard for accuracy comparisons. The use of both 2D and 3D led to a 45.1% reduction in tracing time needed to manually refine automated segmentations (2D: 554 ± 15 min; 2D + 3D: 304 ± 21 min; p = 0.00027; mean difference = 250 min; 95% CI [210.19, 289.81]; Fig. 3 and Additional File 2: Table 1). Using both the 2D and 3D views, manual annotation of the full 3D extent of a nucleus takes approximately 8 min. However, we found that use of both 2D and 3D views was not associated with differences in annotation accuracy relative to the gold standard rater (mean Dice score for 2D: 0.82 ± 0.024; mean Dice score for 2D + 3D: 0.81 ± 0.023; p = 0.86; mean difference = 0.0033; 95% CI [-0.052, 0.059]; Fig. 3 and Additional File 2: Table 1). These findings indicate that combined use of the 2D and 3D views increases speed for manual refinements without sacrificing accuracy in segmentation.

The user survey complemented the case study results, as 2D and 3D views were both found to be useful. Questions focused on the usefulness of editing segmentations in 2D and 3D received respective means of 6.33 (Q1) and 6.83 (Q2) on a 7-point Likert scale, and questions focused on the usefulness of 2D and 3D visualizations received respective means of 5.5 (Q3) and 7.0 (Q4). The region splitting feature was also confirmed to be useful, with a mean of 6.67 (Q6), and questions addressing features related to the region table had an overall mean of 6.63 (Q11-14). Visualizing non-axis-aligned slices in the 2D view supports synchronization of the 2D and 3D views, but scores on the utility of this feature varied, with a mean value of 3.33 and a standard deviation of 2.43 (Q10), perhaps due to artifacts caused by voxel anisotropy. Future work will explore more flexible coupling of the 2D and 3D views to more effectively utilize the strengths of each view.

## Discussion

A user-friendly tool for manual delineation of nuclei in 3D image volumes will greatly accelerate the training of automated recognition algorithms necessary to quantify nuclei in tissue cleared images of the brain. To this end, we have developed Segmentor to make 3D manual annotation easier and more efficient. Segmentor has been tested and iteratively updated based on the feedback of 20 users. Segmentor provides new features that allow the user to parse relevant information and navigate in dense images, automatically split or merge nuclei, keep track of progress during segmentation, and efficiently use both 2D and 3D visual information. While we have designed the tool and demonstrated use cases for segmentation of nuclei from fluorescence microscopy images, Segmentor also can be used to annotate objects from other imaging modalities, such as MRI and CT.

Here, we focus on identifying the borders of the 3D extent of the nucleus rather than using a marker to label one voxel within the nucleus. Though counting applications only require one voxel (or crosshair) within a nucleus to be labeled, labeling the surface or volumetric boundaries of nuclei enables measurements of nuclear shape, facilitates more accurate colocalizations with markers across channels, and allows for evaluation of precision and recall by determining whether an automated segmentation lies within the boundaries of the manually defined nucleus. We also believe that the added information of the nuclear boundaries will provide more useful heuristics to deep learning approaches about contextual features that distinguish the nucleus from the background and possibly other (touching) nuclei [14].

How many manually annotated nuclei are sufficient for training an accurate image segmentation tool using deep learning methods? In recent work [14], 80,692 manually labeled nuclei (from 1,102 images) were used to train a highly accurate 2D segmentation method [29]. Learning 3D nuclei segmentation is more challenging than its 2D counterpart, so it is necessary to develop more complex neural networks (with more parameters), which require larger numbers of training samples for fine tuning the network parameters. Each 3D nucleus is composed of ~5 slices of 2D segmentations at the image resolution used in this work. Thus, our goal is to acquire ~20,000 high-quality manual 3D nuclei annotations using our Segmentor software (comprising ~100,000 2D masks), which will be used to train, validate, and test our neural network model in a tenfold cross validation manner.

The results of our case study suggest that visualization in both 2D and 3D views increases efficiency without impacting accuracy. Because a large number of training samples are needed to train a deep learning-based segmentation model, we expect that the improvement of manual labeling efficiency suggested by our case study will greatly contribute to the performance of automated segmentation software.

Finally, the current approach involves the segmentation of a full 3D image containing 40–400 cells, which still can take 5 to 50 h of manual effort per user, respectively. We expect that as automated initial segmentations improve through training on manually-corrected annotations, time for manual refinement will decrease because fewer manual refinements will be required. Future implementation of a client–server architecture will enable refinement of the machine learning model in conjunction with Segmentor interface enhancements. It will also enable streamlining the ingestion of newly manually annotated training data from Segmentor as input to the machine learning model, and improved machine learning-based initial segmentations as input to Segmentor for manual refining. Additionally, we expect that by chunking these segmentation tasks into smaller units of single cells or clumps of cells, a greater number of users can participate in segmentation simultaneously with reduced overall time commitment. This would allow annotations at a massive scale, through a citizen science approach.

## Conclusions

Segmentor is a freely available software package that increases efficiency of manual refinement in 3D microscopy images. We expect that use of this software will greatly increase the number of training samples and thereby result in higher accuracy of learning-based automated segmentation algorithms, enabling the efficient quantification of brain structural differences at cellular resolution.

## Availability and requirements

- Project name: Segmentor
- Project home page: https://www.nucleininja.org/
- Operating systems: Windows, Mac, Linux
- Programming language: C++ 
- License: MIT
- Any restrictions to use by non-academics: No restrictions

## Supplementary Information

**Additional file 1**. Questionnaire provided to Segmentor users.

**Additional file 2: Table 1**. Table with results of case study.

## Data Availability

The training image dataset analysed during the current study is available from the project webpage: https://www.nucleininja.org/download.

## Abbreviations

2D: Two-dimensional
3D: Three-dimensional
CI: Confidence interval
DSC: Dice score
μm: Micrometers
P15: Postnatal day 15
VTK: Visualization toolkit
