# Supplementary material for: Segmentor: a tool for manual refinement of 3D microscopy annotations
Source: BMC Bioinformatics. 2021 May 22;22:260. doi: 10.1186/s12859-021-04202-8 (PMC8141214; doi:10.1186/s12859-021-04202-8)
Supplement: Supplementary file 2 — Additional file 2: Table 1. Table with results of case study. [file 12859_2021_4202_MOESM2_ESM.docx]

**Supplementary Table 1:** Results of case study to determine accuracy and efficiency of manual refinement when editing and visualizing in 2D only vs. 2D+3D.

|  | **Dice Score**  **p = 0.864** | | **Time in Minutes**  **p = 0.000274** | |
| --- | --- | --- | --- | --- |
| **Image** | **2D only** | **2D + 3D** | **2D only** | **2D + 3D** |
| **1** | 0.787 | 0.797 | 556 | 279 |
| **2** | 0.819 | 0.821 | 545 | 327 |
| **3** | 0.845 | 0.792 | 574 | 314 |
| **4** | 0.814 | 0.842 | 539 | 294 |
| **Average** | 0.816 | 0.813 | 554 | 304 |
